# Rehabilitation management of patients with spinal tuberculosis (Review)

**DOI:** 10.3892/mi.2024.152

**Published:** 2024-04-04

**Authors:** Jaouher Dhouibi, Amine Kalai, Amr Chaabeni, Ahlem Aissa, Zohra Ben Salah Frih, Anis Jellad

**Affiliations:** 1 Department of Physical Medicine and Rehabilitation, Faculty of Medicine, University of Monastir, Monastir 5000, Tunisia; 2 Regional Hospital of Enfidha, Sousse 4030, Tunisia

**Keywords:** spinal tuberculosis, Pott's disease, mycobacterium tuberculosis, spinal cord, compression, pain, rehabilitation, disability

## Abstract

Spinal tuberculosis (ST) is a serious condition and a global health concern, accounting for a significant portion of musculoskeletal tuberculosis cases. It can lead to sever spinal and neurological complications. The management of ST involves a multidisciplinary approach, including medical treatment, surgery and rehabilitation. Rehabilitation is crucial through the course of the disease's and is tailored for each stage according to the patients' complaints, and clinical and functional complications. In the case of neurological issues due to spinal compression, rehabilitation aims at overcoming bed confinement complications, involving mobilization techniques, strengthening exercises and related vesico-sphincter disorders (urodynamics, catheterizing). The role of rehabilitation for the management of pain in patients with ST is based on bracing (restricting movements and relieving the pressure on harmed structures), and analgesic physical means (electrical stimulation and massage techniques). Several rehabilitation options may be used to address musculoskeletal complications. Range of motion exercises, muscle strengthening, and posture and balance correction using sensory perception and proprioception techniques, are commonly involved. Cardiorespiratory reconditioning is required to improve respiratory function, walking ability and cardiovascular endurance. Ultimately, rehabilitation allows for the minimization of disability and the prevention of the loss of autonomy, particularly in elderly patients. The advantage of the rehabilitation approach is its multi-optional characteristics including physical therapy, occupational therapy, ergonomic advices and assistive equipment. Despite its crucial role, rehabilitation remains understudied in the management of ST. Thus, the present mini-review aimed to address the rehabilitation options for the clinical features and complications of ST, according to the course of the disease.

## 1. Introduction

Spinal tuberculosis (ST) or Pott's disease is the infection of the tissues of the spine, particularly the vertebrae and the intervertebral discs, caused by *Mycobacterium tuberculosis* (MT) (1). ST typically originates when MT-infected droplets are inhaled, primarily affecting the lungs, then entering the bloodstream and spreading to other sites of the body. including the spine (2). Therefore, ST is often the consequence of hematogenous dissemination into the dense vascular system of the spongy bone of the vertebral bodies (3).

The incidence of tuberculosis has exhibited an increasing trend over the past decade, reaching eight million new cases per year (4). ST remains a worldwide health concern, accounting for 3 to 4% of tuberculosis cases and 15% of extra pulmonary localizations, and constitutes >50% of all musculoskeletal tuberculosis cases (5). ST may lead to the development of severe spinal and neurological sequelae. In fact, the occurrence of this pathology is often misleading and it is characterized by insidious clinical manifestations, which can lead to a delayed diagnosis and severe complications, such as spinal deformity, paraplegia or tetraplegia (6).

The therapeutic management of ST is based on a multidisciplinary approach, including medical treatment (anti-tuberculosis chemotherapy), on-demand surgery and a specific rehabilitation program (7). Rehabilitation is often a multidisciplinary approach, aimed at restoring and enhancing an individual's physical, mental and social well-being following illness or disability. It involves personalized assessment, goal setting, and a combination of medical, therapeutic and educational interventions provided by a various team of healthcare professionals (physiatrists, physiotherapists and occupational therapists). The main goal of rehabilitation is to improve the quality of life (QoL) and functional abilities of patients (8). Thus, rehabilitation management constitutes a crucial part of the treatment of patients with ST, alongside medical therapy. The implementation of the rehabilitation program is based on a narrow clinical evaluation, seeking the assessment of spinal and neurological complications. Although rehabilitation plays a crucial role in the management of ST, it has not yet received adequate research attention. Thus, analyzing the literature in this field could be of paramount importance. Therefore, the present review aimed to outline the rehabilitation options associated with clinical features, potential complications and the advancement of the disease.

## 2. Rehabilitation of ST neurological complications

A well-defined rehabilitation program should be tailored according to the location of the lesion, the severity of the neurological deficit and the actual functional status. Patients with ST with neurological disorders secondary to spinal cord compression are commonly assessed using the American Spinal Injury Association (ASIA) Impairment Scale (AIS), which provides a standardized method used to determine the level and the severity of the neurological impairment (9). The AIS grading criteria are as follows: Grade A, complete spinal cord injury (SCI) with a complete loss of sensory and motor function in the sacral segments S4 ± S5; grade B, incomplete injury with sensory preservation and a complete loss of motor function below the neurological level, extending to the sacral segments S4 ± S5; grade C, incomplete injury with motor function preserved below the neurological level, but most key muscles below this level have a muscle grade <3; grade D, incomplete injury with motor function preserved below the neurological level, and most key muscles below this level have a muscle grade ≥3; grade E, normal sensory and motor function (no impairment).

Therefore, patients ST with neurological deficit due to spinal cord compression should be managed with the basics of the SCI program (10) which requires the following: Preventive measures of bed confinement complications (adaptive support, pressure ulcer prevention mattress, alternate positions and early progressive verticalization), general peripheral articular mobilization (passive, helped-active or active), core and upper limb strengthening exercises, breathing exercises, encountered spastic muscle posturing and stretching, and the assessment of any vesico-sphincter disorders in order to avoid damage to the upper urinary tract and to preserve renal function. In fact, some cases may require intermittent catheterization training to maintain appropriate bladder emptying. In the latter situation, a physiatrist educates and supports the patient or their caregiver in performing clean intermittent catheterization. The physiatrist emphasizes various precautions, including adequate hand hygiene to reduce the risk of urinary tract infections, ensuring comfortable positioning, and gently inserting the lubricated catheter to prevent urethral irritation or injury. Initially, the physiatrist conducts the procedure with detailed explanations, and the patient then carries it out under the doctor's supervision. Regular follow-up appointments are necessary to assess the patient's proficiency and provide ongoing assistance to ensure the continued success of the procedure.

For example, in the case of ST with L1 spinal cord compression and an AIS grade A, the prognosis is expected to be severe, resulting in paraplegia with a complete loss of motor and sensory capabilities below the L1 neurological level. The patient will be exposed to frequent complications, such as deep vein thrombosis and thromboembolic events, pressure sores and decubitus ulcers. Additionally, they may experience disuse-induced muscle atrophy, urinary tract infections, urinary retention, bladder dysfunction and constipation. The rehabilitation management is primordial in this case, consisting of passive mobilizations of the lower limbs and the prescription of compression socks to improve lower limbs circulation, specific mattress, alternating positions, and early progressive verticalization for ulcer prevention, and intermittent urinary catheterization is vital to prevent urinary infections and upper urinary tract complications. This management will be tailored according to clinical improvement.

The rehabilitation program may be challenging in the case of ST, involving the cervical spinal level. In the case of complete tetraplegia, particularly when cervical stages above C6 are involved and the case is categorized as AIS grade A, bed confinement complication preventive measures are of paramount importance. Special attention should be paid to alternating positions, correct patient positioning, breathing exercises and chest physiotherapy, as well as urinary catheterization. In fact, the risk of ulcers, respiratory complications, joints stiffness and deformities, and urinary complications is higher in tetraplegic patients. Thus, the paralysis of upper and lower limbs implicates the constant need for external assistance to perform these measures. Furthermore, these patients are characterized by dysautonomia complications, including cardiovascular issues, impaired temperature regulation and autonomic dysreflexia. Thus, regular monitoring by a healthcare professional is crucial to tailor treatment.

Despite the ST level, in patients with AIS grade D or E, the rehabilitation program will focus mainly on flexibility and strengthening exercises, proprioception techniques, balance and gait tasks, and cardiorespiratory reconditioning.

## 3. Rehabilitation of pain associated with ST

Pain is the main complaint of patients with ST. Of note, 90 to 100% of patients complain of back pain (11). The immobilization of the affected spinal segment with adequate equipment (a corset in the case of lumbar or dorsal involvement and a cervical collar in the case of cervical spine involvement) and in some cases, strict bed rest, are primordial measures used to decrease the intensity of pain (12). In fact, bracing offers the advantage of combining several targeted objectives which are restricting movement, relieving pressure, promoting the healing and relieving the weight on the involved spinal region. Furthermore, bracing is of paramount importance following eventual surgery, providing post-operative support. However, attention should be paid to the occurrence of decubitus complications potentially worsening the prognosis. Thus, early and progressive physiotherapy could be helpful to prevent such complications (13).

In addition to painkillers, physical means, particularly transcutaneous electrical nerve stimulation (TENS), are paramount, mainly in the management of neuropathic pain by activating the gait control at the spinal level, therefore modulating painful pathways. For nociceptive pain, TENS with other heat physical means, such as ultrasounds and infrared light may attenuate patients' pain by acting on superficial and subcutaneous anatomical structures and reducing local pain mediators. However, before employing TENS, it is imperative to observe several precautions to guarantee its safe and effective application. A physiatrist should rule out any medical conditions, such as a pacemaker, heart condition, epilepsy, skin diseases or sensory impairment that contraindicate the application of TENS. In addition, the presence of a physiotherapist is obligatory during the TENS session to ensure vigilant monitoring for skin sensitivity, irritation, burns, or allergic reactions. Therefore, adhering to precautions diligently and promptly addressing any side-effects is indispensable for ensuring the safe and effective utilization of TENS therapy in pain management. Additionally, joint mobilizations and musculo-tendinous stretching are useful for relieving musculo-skeletal pain (12,13).

## 4. Rehabilitation of ST musculoskeletal, postural, balance and gait complications

Musculoskeletal complications, essentially joint stiffness, apart from upper and lower limb and spinal muscular weakness should be managed in order to prevent the increased risk of possible bone destruction and deformity, and to promote functional outcomes. The rehabilitation program may involve the following: A range of motion exercises to all joints of the upper and lower limbs to maintain or improve joint mobility, and prevent muscular contractures and hypotrophy (12). Additionally, the strengthening of core and upper and lower limb muscles through isometric and resistive exercises using manual resistance initially, and weight cuffs at a later stage, allows for overcoming muscular weakness.

Furthermore patients with ST are at a high risk of developing postural abnormalities, particularly spinal kyphosis (14). Thus, the education of patients on proper body mechanics and posture, alongside specific rehabilitation techniques are paramount for minimizing the risk of spinal deformities, particularly kyphosis and adjusting postural issues (15). These techniques may comprise active the axial self-stretching of the spine, thoracic expansion work, repositioning and the active stretching of the anterior pectoralis and lower limbs muscles, and the strengthening of the spinal extensor muscles with isometric, concentric and eccentric dynamic exercises. The strengthening program should be progressive, beginning with isometric contractions (where the muscle contracts but does not change length, and there is no movement at the joint; the force generated by the muscle is equal to the applied resistance), and subsequently progressing to concentric contractions (where the muscle contracts while shortening, and the force generated is greater than the applied resistance), and ultimately to eccentric contractions (where the muscle contracts while lengthening, and the force generated is less than the applied resistance). These exercises should be performed under the supervision of a physical therapist to ensure these are commenced with appropriate weight, gradually increasing intensity, and monitoring muscle fatigue to prevent muscle soreness and damage. Patients may receive instructions and ergonomic advices to promote and maintain an appropriate posture during daily activities.

Neurological and musculoskeletal impairments may lead to balance and proprioception disorders, particularly in the elderly and those with morbidities who are at a high risk of falls and loss of autonomy. Exercise-based rehabilitation aiming at the improvement of sensory perception and proprioception in affected limbs enables patients to regain stability, enhance coordination, restore normal walking patterns and improve mobility. This, in turn, helps reduce the risk of falls and injuries, particularly in elderly patients. Exercises focus on walking ability and proprioception, according to the neurological damage and the degree of recovery. They may involve assisted walking with the aid of a physical therapist and mobility aids, such as a walker or parallel bars, functional electrical stimulation to stimulate leg muscles and promote walking patterns, and seated leg extensions targeting the quadricep muscles and improve knee stability (16). In order for patients to maintain their autonomy, further assistive equipment, such as crutches, walking frames and wheelchairs may be helpful.

Additionally, patients are prescribed postural reprogramming, involving exercises to correct seated and standing postures, the stretching of tight muscles, biofeedback training utilizing devices or mirrors for visual feedback on posture alignment, and breathing exercises aimed at promoting relaxation and proper spine alignment. Patients are re-trained to perform progressive resistance training with hand resistance, resistance bands and free weights, and functional training focusing on daily living activities (16). Daily activities and QoL may be promoted by physical therapy, occupational therapy, ergonomic advices and the patient's environmental adaptation.

## 5. ST cardiorespiratory rehabilitation

Patients with ST commonly have limited activity during the acute phase of the disease with frequent cardiorespiratory deconditioning. In this stage, patients should commence cardiorespiratory rehabilitation, even in bed confinement, through breathing exercises, which include pursed lip breathing and thoracic expansion exercises in order to improve respiratory function. Furthermore, respiratory muscle training techniques, including the use of incentive spirometers or respiratory muscle trainers are useful for strengthening breathing muscles and enhancing respiratory endurance. Flexibility exercises incorporated in stretching routines targeting joint stiffness and muscle tightness may enhance overall functional capacity (17).

Furthermore, cardiorespiratory rehabilitation will involve working on walking ability, cardiovascular endurance and reconditioning with a progressive schedule. Exercises programs may begin with progressive walking with the aid of a physical therapist and mobility aids such as a walker or parallel bars. Aerobic exercises may involve arm ergometry, such as arm bike or hand cycle training (combined effects on cardiovascular fitness and upper body strength), treadmill, staircase climbing, stationary bicycle and aquatic therapy (aerobic exercises in water). At an advanced stage, fast walking, jogging and running with interval training (alternating between periods of high intensity exercise and rest or lower intensity exercise) may be used according to the capacities of the patient (12).

**Figure 1:**
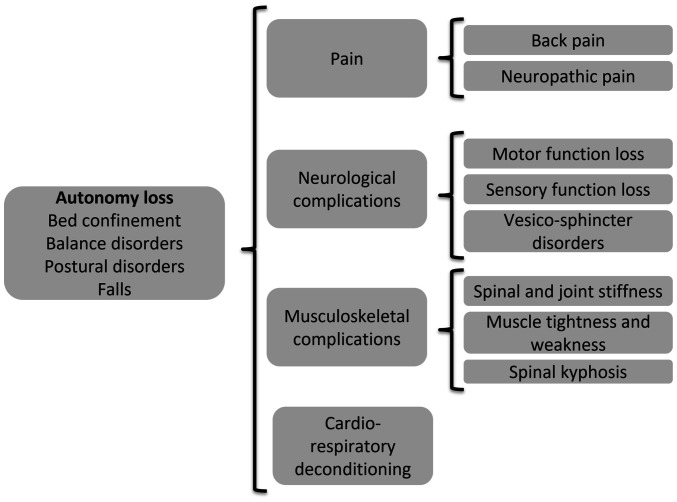
Factors contributing to the loss of autonomy in patients with spinal tuberculosis.

**Figure 2:**
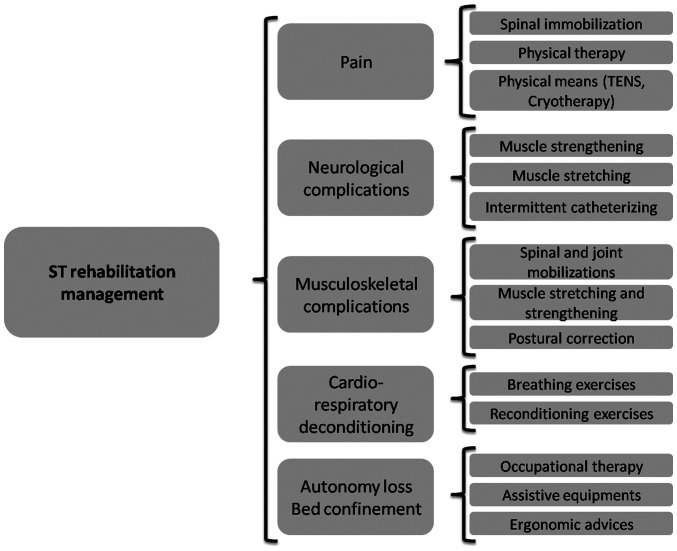
Rehabilitation interventions depending on the impairments of patients with ST. ST, spinal tuberculosis; TENS, transcutaneous electrical nerve stimulation.

In summary, the combination of several symptoms and complications of ST may result in the loss of autonomy and bed confinement, accompanied by several related complications (Fig. 1). The rehabilitation management of patients with ST offers multiple options, targeting the main disabling symptoms and clinical abnormalities (Fig. 2).

## 6. Conclusion and future perspectives

Rehabilitation plays a crucial role in the management of patients with ST, addressing a spectrum of complications, including neurological, pain, musculoskeletal, postural, balance, gait and cardio-respiratory issues. Hence, it aims at primarily minimizing disability, regaining autonomy and improving the QoL of patients. Rehabilitation programs should be tailored according to the impairments of patients, such as muscle weakness, spine and joint stiffness, postural disorders, and cardio-respiratory deconditioning. Despite the plethora of rehabilitation techniques with notable efficacy in the management of ST, there remains a lack of well-codified programs tailored to its stages and complications. Further research is imperative, particularly to analyze optimal bracing duration and verticalization delay. Robotics and artificial intelligence hold promise for significantly enhancing the efficacy of rehabilitation in this domain.

## Data Availability

Not applicable.
